# The Mediating Effects of Social Support and Locus of Control on the Relationship between Post-Traumatic Stress and Depressive Symptoms in a Jamaican University Sample

**DOI:** 10.4172/2167-1044.1000194

**Published:** 2015-07-31

**Authors:** Azizi A. Seixas, Caryl James, Girardin Jean-Louis, Mark Butler, Ferdinand Zizi, Alex Gardner

**Affiliations:** 1 Center for Healthful Behavior Change, Department of Population Health, NYU School of Medicine, NY, USA; 2 The University of the West Indies, Mona, Jamaica

**Keywords:** Depression, Posttraumatic stress symptoms, Young adults, Social support, Locus of control

## Abstract

**Background:**

The increasing rate of comorbid posttraumatic stress and depressive symptoms among young adults presents a unique symptom presentation and challenges to treatment. The current study examined psychosocial barriers--external locus of control-- and facilitators-- social support-- in the posttraumatic stress and depressive symptoms association.

**Methods:**

The current cross-sectional study was conducted among 701 Jamaican university participants, ages 18–30 years. Participants completed self-report measures of general demographic information as well as target variables which include the CES-D-10, Sense of control (external and internal locus of control), Short screening scale for DSM-IV posttraumatic stress disorder and social support measures.

**Results:**

Majority of the sample was female (76.2%; n=534); and slightly more than half of the sample self-identified as Black/African ancestry (59.7%). External locus of control (LOC) partially mediated the relationship between posttraumatic stress and depressive symptoms, external locus of control (LOC) had a greater mediation magnitude than social support in the posttraumatic stress-depressive symptoms association (Indirect Effect=0.133, 95% CI-0.075–0.211). In post-hoc analyses women appeared more highly traumatized than their male counterparts (14.3%, χ^2^ =8.032, p=0.005). The sub-sample of highly traumatized individuals reported higher levels of depression, posttraumatic stress symptoms, external LOC, and lower levels of social support and internal LOC than did individuals with lower levels of trauma.

**Conclusion:**

Contrary to previous research, our findings indicate that external LOC partially mediated the relationship between posttraumatic stress and depressive symptoms among a Jamaica university sample more so than social support. These findings therefore suggest that psychosocial treatments should consider locus of control focused interventions or skill building for young adults who suffer from posttraumatic stress and depressive symptoms.

## Introduction

The global prevalence of depression among young adults (late teens to mid-thirties) have been on the rise [1], and recent evidence shows that depression has become considerably more prevalent in developing and middle-income countries, like Jamaica [2,3]. One study showed that 40% of university-age students (late teens to mid-thirties) scored in the clinically depressed range [4], which is higher when compared to a nationwide survey conducted by American College Health Association–National College Health Assessment which reflects 30% [5]. Though several factors have been implicated in the development and maintenance of depressive symptoms [6,7], the high prevalence of comorbid posttraumatic stress symptoms or trauma-related symptoms may mean that the one causes the other or perhaps the two are inextricably linked either by shared etiologies [8–10]. Regardless of what causes the comorbidity, research has shown that such comorbidity complicates treatment, more than instances where there is a single posttraumatic stress or depressive symptoms [11]. Although there is clear evidence that posttraumatic stress and depressive symptoms are linked, there is some ambiguity regarding how they might co-occur.

The comorbidity of post-traumatic stress and depressive symptoms is well established [11–13]. Depression is considered the most comorbid psychiatric disorder with post-traumatic stress disorder (PTSD) and is often couched as part of the PTSD sequelae [12]. Among university students, the prevalence of comorbid PTSD and depression varies between more than a half of the sample to the majority of the sample- 51.6% [12] 62% [14,15] and 92% [16]. Despite the foregoing evidence, a clear mechanism as to how and why posttraumatic and depressive symptoms are interrelated is lacking. Of the many factors that might explain comorbid posttraumatic stress and depressive symptoms, two schools of thought have received the most traction. These include: 1) a genetic link, established by twin studies [17,18], where Fu and colleagues found that the association between PTSD and depression was largely genetic, as 19% of the variance in depression could be explained by PTSD; and 2) a cognitive link, where intrusive memories and thoughts, overgeneralization [19], negative thinking, self-blame [20], ruminative thinking [21], and thoughts of helplessness [22–26] coupled with stimulus and emotional avoidance, numbing, increased emotional arousal and re-experiencing, are all characteristic of comorbid PTSD-MDD. Of note, individuals with comorbid posttraumatic stress and depressive symptoms have difficulty trusting their environment and believe they have little to no control over their lives as it is determined by fate, circumstances or by someone else. These beliefs prevent the individual from using adequate coping resources to ward off psychological harm, thus making them susceptible to further psychological problems [11].

Understanding comorbid posttraumatic stress and depressive symptoms is important for treatment since the presence of the two presents a more debilitating clinical picture as well as complicates treatment [27]. Psychosocial interventions targeting comorbid posttraumatic stress and depressive symptoms indicate that social support from friends, family and community [28,29] plays a major role in symptom reduction and partially protects the individual from further psychological problems, like depressive symptoms [28–34]. Perceived frequency of social support is considered more effective at reducing severity of psychological symptoms, compared with the quantity, variety, or availability of familial or friendship support [30–34]. Amidst the aforementioned benefits, there is no clear evidence that social support addresses feelings of helplessness, depressive cognitions, and depleted resilient coping skills, common psychological symptoms of comorbid posttraumatic stress and depression [35,36].

Being able to deal with helplessness, depressive cognitions, and depleted coping resources are a function of an individual’s locus of control (LOC) [37,38]. LOC can be internally or externally driven and, depending on which is used, may result in symptom reduction or exacerbation [39]. Individuals who are internally driven tend to be protected from undesirable psychological outcomes, as they are able to adjust and tap into their reservoir of coping—a sign of internal resilience [40]. Externally driven individuals on the other hand, leave their fate to chance, luck, or other external forces, which may lead to higher levels of depression, anxiety, and lower levels of life satisfaction. Recent evidence indicates that externally driven individuals with a trauma history are at an increased risk for PTSD, which can cascade into other psychological comorbidities, therefore making treatment more difficult [22–26,37].

The current paper investigates how psychosocial factors, such as social support and external locus of control, mediate the relationship between post-traumatic stress and depressive symptoms. Specifically, individuals with posttraumatic stress symptoms and externally driven attributions of their symptoms (reflected in LOC) may yield greater pathology, in spite of protective factors such as social support. We hypothesized that both external LOC and social support would mediate the relationship between posttraumatic stress and depressive symptoms but were uncertain about the degree by which they would.

## Methods

### Procedures and participants

The current observational study utilized data from the Jamaica sample of the larger Health and Behavior Survey of university students, conducted in 26 countries (N=19264). The sample consisted of 701 students who were between the ages of 18–30. Investigators obtained ethical approval from the University Hospital of the West Indies and the University of the West Indies Faculty of Medical Sciences Ethics committees. After gaining ethical approval, lecturers for the selected classes were contacted and a time slot was given for the study to be introduced to their respective classes. Participants who agreed to participate in the study were given consent forms and self-administered questionnaires. After completion of the survey, participants were then ushered to an area of the room where their anthropometric measurements were taken.

### Measures

#### Depression

Depressive symptoms were measured using the Centre for Epidemiologic Studies Depression Scale – short version (CES-D –10). The CES-D-10 is the shortened easy to administer, reliable and valid version of the 20-item CES-D questionnaire developed by Anderson et al. (1994). It assesses depressed mood over the past week, and has been shown to be able to correctly identify clinical depression in child, adolescent and adult samples. Each item is measured on a 4-point likert scale, ranging from rarely (less than one day=0) to most or all of the time (5 – 7 days=3) [38,39]. Examples of items include: “I felt depressed, I felt lonely, My sleep was restless… Scores on this measure range from 0 to 30, with a score of 10 or more indicating the presence of depressive symptoms. The CES-D-10 has demonstrated sound reliability with internal consistency scores ranging from Cronbach α of 0.71 to 0.85 [38,39]. Cronbach’s α for the current study was 0.76.

#### Locus of control (LOC)

We measured participants’ sense of control by adopting two concepts: Personal Mastery and Perceived Constraints that were obtained from the Midlife in the United States (MIDUS]. These subscales have demonstrated good internal validity: Personal Mastery Cronbach’s α was 0.70 and Personal Constraints was 0.86 [36]. Of these two categories (Personal Mastery and Perceived Constraints), a total of 3 questions each were selected from the original construct. A sample question for Personal Mastery subscale is “I can do just about anything I really set my mind to” and a sample question for the Perceived Contraints subscale is “I often feel helpless in dealing with the problems of life”. Each item is measured on a 5-point likert scale ranging from 1(strongly disagree) to 5 (strongly agree). Three items were reversed scored (“There is little I can do to change many of the important things in my life”; “I often feel helpless in dealing with the problems in my life”; and “I have little control over the things that happen to me”) and the mean of the items computed. A higher score reflects greater personal mastery. In our study the Cronbach’s α was 0.62.

#### Short screening scale for DSM-IV posttraumatic stress disorder

This is a seven-item scale that assesses post-traumatic stress symptoms (41). Participants were asked to indicate ‘yes’ or ‘no’ on five of the symptoms assess avoidance and numbing symptoms and the other two assesses hyperarousal symptoms. A score of 4 or greater on this scale defined positive cases of PTSD with a sensitivity of 80%, specificity of 97%, positive predictive value of 71%, and negative predictive value of 98%. Therefore, it can be said that if a person indicated yes to four or more of these questions, a PTSD diagnosis is likely [41]. In our study the Cronbach’s α was 0.76.

#### Social support

Social support was measured by three items drawn from the Social Support Questionnaire [42]. Each item is measured on a 4-point likert scale from completely true to completely false. The questions were selected in a manner similar to previous studies [43]. To assess perceived tangible and emotional support: “If I were sick and needed someone to take me to a doctor I would have trouble finding someone” (reversed); “I feel that there is no one I can share my most private concerns and fears” (reversed); and “I feel a strong emotional bond with at least one other person”. Cronbach’s α for this sample was 0.50.

#### High level of traumatization

This post-hoc scale is a dichotomous measure created by combining clinically significant scores on both the short screening scale of PTSD) a proxy for traumatic symptoms and reactions which include avoidance, numbing, and hyperarousal behaviors) and another measure asking individuals whether they experienced five types of traumatic events by indicating ‘yes’ or ‘no’. Individuals who reported one or more traumatic events in their lives and reported a significant number of posttraumatic stress symptoms (4 or more) on the short screening measure were classified as having a high level of traumatization. The goal of this measure was to select a sub-sample of the populations who had reported experiencing traumatic events in their lives and also reported clinically significant levels of posttraumatic stress symptoms.

### Data analysis

The data were analyzed using IBM SPSS Statistics for Windows (Version 20.0, Armonk, NY, USA). Gender-based comparisons were made using independent samples t-tests. A series of bivariate and multivariate regression analyses were conducted to examine relationship between depression (dependent variable), gender, social support (independent variable and mediator), posttraumatic stress symptoms (independent variable), and Locus of Control (independent variable and mediator). Moderation analyses were also conducted to examine whether gender affected the relationship between posttraumatic stress symptoms, Locus of Control and Depression. Finally, we conducted mediational analyses to examine whether Locus of Control mediates the effect of posttraumatic stress symptoms on depressive symptoms. All mediation analyses were conducted using the PROCESS macro [44]. This macro generates coefficients using ordinary least squares regression and provides estimates for direct and indirect effects in mediation with bootstrapped confidence intervals. To demonstrate model significance, we reported R-squared values for both steps of the mediational model as well as the R-squared value for the direct effect. Bootstrapped 95% CI values of the indirect effect of the independent variable (IV), posttraumatic stress, on the dependent variable (DV), depressive symptoms, via the mediator as well as the direct effect of the IV on the DV were reported and evaluated.

In post-hoc analyses we examined the sub-sample of highly traumatized individuals. We examined the gender differences in highly traumatized individuals and examined whether being a member of the highly traumatized sub-population predicted depression symptoms when controlling for locus of control, social support and covariates.

#### Covariates

We adjusted for age, perceived social support, and BMI in our regression analysis. For the BMI, students were weighed and measured by trained researchers using standardized protocols [45]. Standing height was measured to the nearest 0.1 cm without shoes, using a stadiometer. Participants wearing light clothes were weighed to the nearest 0.01 kg on a load-cell-operated digital scale which was first calibrated using a standard weight and re-checked daily [41]. Body mass index (BMI) was calculated as weight in kg divided by height in meters squared.

## Results

### Descriptive statistics and characteristics of sample

The current study consisted of 701 participants from a Caribbean university site. Ages ranged from 18 to 30 years (M=20 ± 2.03); female students represented the majority of the sample (76.2%; n=534); and slightly more than half of the sample self-identified as Black/African ancestry (59.7%), 31% as mixed race, 3.5% as Indian or Asian, 0.1% as white, and 5.7% as Other. Despite having all levels of undergraduates participating in the study, the majority were in their first year of studies (74.2%) and the least in their fourth year of studies (1.3%). Almost all of the students indicated that they were not married (98.7%) and slightly more than half indicated that they lived at home with their parents (59.3%).

The sample reported average depressive symptoms, which were below the cutoff for clinical significance (mean=9.21, SD=5.43). The sample also reported slightly more than two posttraumatic stress symptoms on average (mean=2.28, SD=0.205) and less than 1 traumatic event (mean=0.041, SD=0.078). The sample also displayed high levels of social support (mean=9.72, SD=1.89). Of the traumatic events, being forced to have sex was the most reported traumatic event, while being diagnosed with HIV was the least reported.

Gender-based analysis of target variables indicates that women reported significantly higher levels of depression and social support than did men (p=0.007 and p=0.043 respectively) (Table 1). Men and women did not differ in terms of reported posttraumatic stress symptoms or Locus of Control scores.

**Figure 1:**
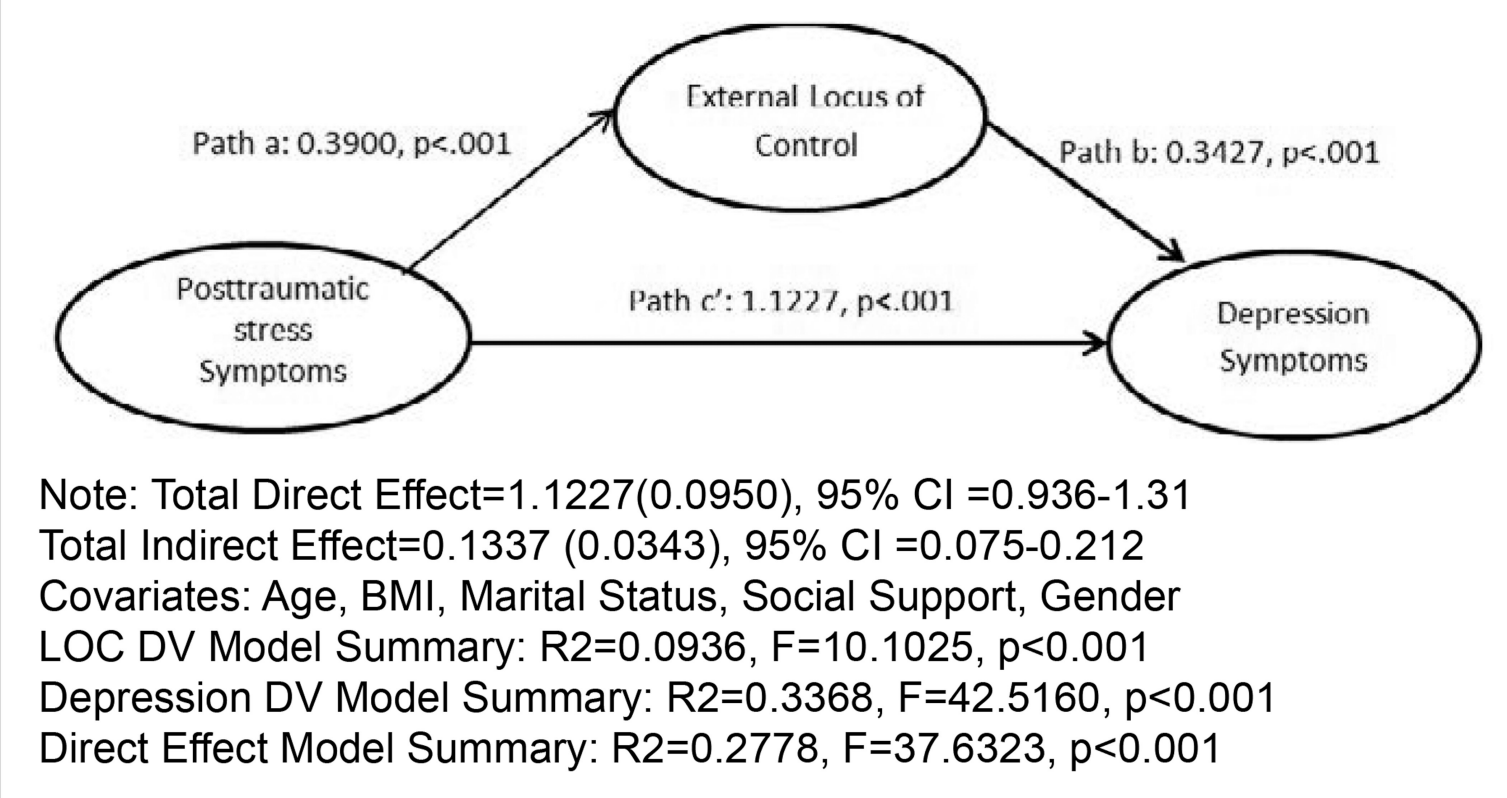
The mediating effect of PTSD External Locus of Control on the association between PTSD and Depression. Note: Total Direct Effect=1.1227(0.0950), 95% CI =0.936–1.31 Total Indirect Effect=0.1337 (0.0343), 95% CI =0.075–0.212 Covariates: Age, BMI, Marital Status, Social Support, Gender LOC DV Model Summary: R2=0.0936, F=10.1025, p<0.001 Depression DV Model Summary: R2=0.3368, F=42.5160, p<0.001 Direct Effect Model Summary: R2=0.2778, F=37.6323, p<0.001

Examining the possible relationship among trauma, locus of control and depression in a context of mediation showed that there appears to be some evidence that locus of control may partially mediate the effect of PTSD symptoms on depressive symptoms (Figure 1). Though PTSD directly symptoms affected depressive symptoms (Direct Effect=1.123, 95% CI=0.936–1.31), external locus of control partially mediated this effect (Indirect Effect=0.133, 95% CI-0.075–0.211). This indicates that for every one unit increase in symptoms of PTSD (i.e. an additional symptom), there is a 1.123 unit increase in depression directly due to posttraumatic stress symptoms and a 0.123 unit increase in depression via the relationship between posttraumatic stress symptoms and External Locus of Control. We then examined a multiple mediation model examining the indirect effect of posttraumatic symptoms on depression (CES-D scores) mediated by both social support and external locus of control. This analysis showed small but significant indirect effects of posttraumatic symptoms on depression mediated by the both social support and locus of control (Indirect Effect= 0.0519 and 0.1610 respectively). Once again the direct effect of posttraumatic stress symptoms on depression was greater than the total indirect effect (Direct Effect=1.1227).

We then conducted post-hoc analyses examining the sub-population of the sample who reported one or more traumatic events and had clinically significant levels of PTSD symptoms (>4). For the purposes of discussion, this sub-population was labeled as highly traumatized. We found that high levels of trauma occurred in roughly 12% of the sample (N=86). In terms of gender differences, a significantly higher percentage of women had high levels of trauma (14.3%, X^2^=8.032, p=0.005) (Table 2). We then examined whether levels of depression, social support, PTSD symptoms, and locus of control differed between individuals who were highly traumatized and individuals who were not. Our results showed that individuals with high levels of trauma had greater depressive symptoms, posttraumatic stress symptoms and External Locus of Control (p<0.001). We also found that highly traumatized individuals had lower levels of social support and internal locus of control (p<0.05) (Table 3).

We then ran a linear regression predicting depressive symptoms using high levels of trauma, external locus of control and female gender as predictors. We found these to be significant predictors of depressive symptoms even after adjustment for covariates (age, BMI, marital status and social support). Neither trauma symptoms nor locus of control significantly interacted with gender in predicting depressive symptoms (p>0.1) (Table 4).

## Discussion

**Figure 2:**
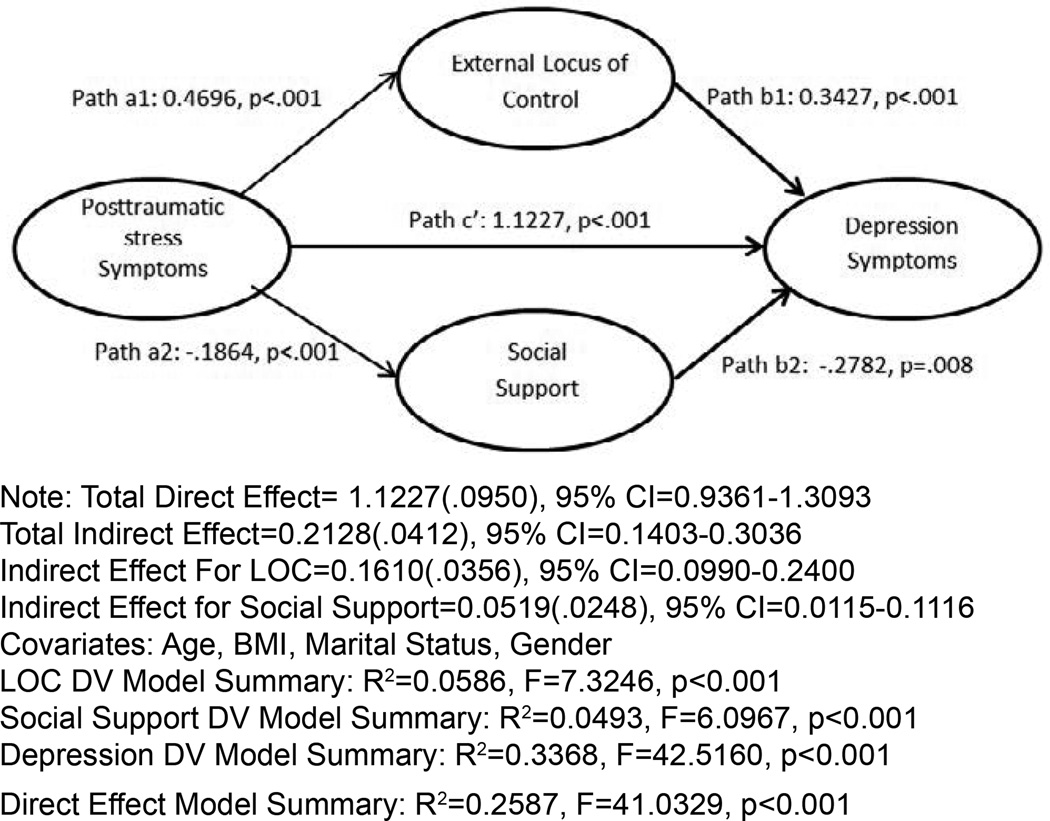
The mediating effect of external locus of control and social support on the association between PTSD and depression. Note: Total Direct Effect= 1.1227(.0950), 95% CI=0.9361–1.3093 Total Indirect Effect=0.2128(.0412), 95% CI=0.1403–0.3036 Indirect Effect For LOC=0.1610(.0356), 95% CI=0.0990–0.2400 Indirect Effect for Social Support=0.0519(.0248), 95% CI=0.0115–0.1116 Covariates: Age, BMI, Marital Status, Gender LOC DV Model Summary: R^2^=0.0586, F=7.3246, p<0.001 Social Support DV Model Summary: R^2^=0.0493, F=6.0967, p<0.001 Depression DV Model Summary: R^2^=0.3368, F=42.5160, p<0.001 Direct Effect Model Summary: R^2^=0.2587, F=41.0329, p<0.001

Our study makes three significant contributions to the literature. First, we observed that external Locus of Control partially mediated the relationship between posttraumatic stress and depressive symptoms. Second, external Locus of Control had a greater mediation magnitude than social support in the relationship between post-traumatic stress and depression, as evidenced by greater indirect effects (Figure 2 path a1, b1, a2 and b2). Third, post-hoc analysis demonstrated that women were more highly traumatized than their male counterparts, and highly traumatized individuals reported higher levels of depression, PTSD symptoms, external Locus of Control, and lower levels of social support. Some of these findings are consistent with extant research [22,46–48] while other findings add new theoretical and clinical insights about psychological and cognitive correlates of comorbid PTSD and major depressive disorder (MDD). These correlates may have significant impact on the etiology and treatment of comorbid post-traumatic stress disorder and depression.

The finding that external Locus of Control partially mediated the relationship between posttraumatic stress and depressive symptoms might be explained by two theories. First, individuals with high levels of trauma may have high external LOC perhaps before the traumatic event or may have developed heightened external LOC after the traumatic event because they no longer feel they have control over their lives and their ability to navigate in their world [22–26,37]. Second, since individuals with high levels of posttraumatic stress symptoms are more likely to report higher external LOC, they are more vulnerable to develop depressive beliefs and thoughts, which engender feelings of helplessness and hopelessness [23]. Such findings can have a significant impact on treatment whereby individuals with high external LOC are likely to become overly dependent on sources of social support such as a therapist, which can undermine treatment [23].

Social support is a critical component in the posttraumatic stress and depression association. Prior studies indicate that social support likely buffers a traumatized individual from developing depressive symptoms. Other studies have found that social support may generally protect an individual from serious mental illness or facilitate greater recovery and symptom reduction from comorbid posttraumatic stress and depression [30–34]. However, results have been mixed as social support may only be partially protective. Our finding that external LOC is a stronger mediator than social support, albeit opposite effects, might explain why social support does not fully explain recovery and symptom reduction of comorbid posttraumatic stress and depression. Evidence from the current study suggests that external LOC, or the feeling of not being in control of one’s life, regardless of social support, may maintain depressive symptoms and reduce receptivity to of the benefits of social support. This is consistent with the idea that perceived lack of control evidenced by high levels of external LOC is linked to feelings of helplessness, hopelessness, and diminished will power [47]. The treatment implication of this finding is that it may shed light on why social support may not work because the individual may have a belief system or cognitive schema that is in line with external LOC, which may impede treatment success.

Although there did not appear to be to be dramatic differences in posttraumatic symptoms between genders or any significant interaction effects between gender and either LOC or posttraumatic symptoms in predicting depression, our post-hoc analysis showed that: a) women may have overall higher levels of trauma than men and b) individuals who are highly traumatized report greater levels of depression, posttraumatic symptoms, external LOC but lower protective factors such as internal LOC and social support highlight limitations and raise concerns that future projects will have to address.

## Limitations and Future Recommendations

While this study provides useful information on a portion of Jamaican university, there are several limitations. Firstly, sole reliance on self-report measures as the primary means to assess individual’s control-related beliefs, depressive, and posttraumatic stress symptoms may be subject to response bias. A second limitation relates to the validity of the scales used for locus of control. While this measure is moderately reliable, future studies should include standardized measures of locus of control to assess control-related beliefs. Using a longer, more psychometrically sound measure of locus of control orientation may be more sensitive in detecting gender differences. The last limitation of the current study pertains to our classification of high traumatization based on the presence of both an reported traumatic event and significant levels of posttraumatic stress symptoms. However, it is possible that individuals may have been traumatized by an event that was not measured in the current design. This is a potential limitation in our classification of the subpopulation with High Levels of Trauma.

It is recommended that future studies include an investigation of the number and degree of stressors that contribute to university students’ posttraumatic stress and depressive symptoms. Including this information in future studies could provide a better understanding of the etiology of comorbid posttraumatic stress and depressive symptoms among Jamaican young adults, as well as the individual and combined effects of stressors and cognitions on the development of these symptoms. Similarly, replication of this study’s result on different samples would prove to be advantageous by exploring the role that age and socio-economic status play in the relationships with posttraumatic stress, external LOC, social support and depression. Since locus of control occurs on a continuum and young adults experience cognitive changes based on situational demands [43], use of a prospective or longitudinal design will prove useful in determining if manipulation of LOC belief reduces likelihood of developing depression, or reduces severity of posttraumatic stress or depressive symptoms. Future studies should investigate which posttraumatic stress symptoms are related to locus of control and depression. Lastly, future studies should examine high levels of trauma with multiple measurements (or well validated measures).

## Conclusion

Posttraumatic stress and depressive symptoms make up a large portion of the growing rate of psychopathology among young adults globally, especially in developing and middle income countries. In sum, our study found that while social support buffers the relationship between posttraumatic stress and depressive symptoms in a predominantly black Caribbean population, it appears that external locus of control has a greater effect than social support. Based on these findings, treatment should address feelings of control, a function of external LOC, in treating individuals who exhibit posttraumatic stress symptoms because they are likely to develop depressive symptoms. As evidenced by our findings, the presence of posttraumatic stress symptoms and external LOC increases the likelihood of depressive symptoms even with social support. Treatment should take a two-pronged approach that includes cognitively restructuring helpless and hopeless thoughts and providing adequate social support. Evidence-based treatments, such as cognitive-behavior therapy and/or pharmacological approaches should prove useful in helping individuals come to a greater sense of control over their circumstances and engage in problem-solving strategies rather than externalizing blame. Understanding the impact control-related beliefs have on depression engendered by PTSD symptoms would enable health care professionals to provide more targeted treatment.

## Figures and Tables

**Table 1:** t-tests comparing major predictors and outcomes between genders.

Variable	Women	Men	Mean Difference	p-value
Depression	9.52 (5.64)	8.13 (4.85)	1.38 (0.51)	0.007*
Posttraumatic stress Symptoms	2.36 (2.07)	2.04 (1.99)	0.32 (0.08)	0.078
Total Locus of Control Scale (6 items)	13.05 (4.21)	1255 (3.89)	0.49 (0.37)	0.180
External Locus of Control Scale (3 items)	7.34 (2.90)	6.89 (2.67)	0.44 (0.25)	0.081
Internal Locus of Control Scale (3 items)	12.08 (2.52)	12.05 (2.53)	0.02 (0.22)	0.918
Social Support	9.80 (1.86)	9.46 (1.94)	0.34 (0.17)	0.043*

Note: Depression=for Epidemiologic Studies Depression Scale (CES-D); Posttraumatic Stress= Short screening scale for DSM-IV posttraumatic stress disorder.

* p<0.05;

** p<0.001

**Table 2:** Comparing frequency of high levels of traumatization between men and women (Post-hoc analysis).

Variable	Category	Highly Traumatized		Total
		No	Yes	
Gender	Female	455 (85.7%)	76 (14.3%)	531 (100.0%)
	Male	156 (94.0%)	10 (6.0%)	166 (100.0%)
Total		611 (87.7%)	86 (12.3%)	697	

Pearson Chi-Square=8.032, p=0.005

**Table 3:** T-tests comparing major predictors and outcomes between high trauma (Post-hoc analysis).

Variable	Not High Trauma	High Trauma	Mean Difference	p-value
Depression	8.63 (5.10)	13.86 (5.69)	−5.23 (0.64)	<0.001
Posttraumatic Stress Symptoms	1.93 (1.87)	5.11 (1.01)	−3.17 (0.22)	<0.001
Total Locus of Control Scale (6 items)	12.67 (4.01)	14.99 (4.56)	−2.32 (0.49)	<0.001
External Locus of Control Scale (3 items)	7.07 (2.79)	8.64 (2.94)	−1.57 (0.34)	<0.001
Internal Locus of Control Scale (3 items)	12.18 (2.49)	11.42 (2.49)	0.75 (0.30)	0.013
Social Support	9.81 (1.83)	8.99 (2.17)	0.82 (0.23)	<0.001

Note: Depression=Center for Epidemiologic Studies Depression Scale (CES-D); Posttraumatic Stress=Short screening scale for DSM-IV posttraumatic stress disorder.

* p<0.05;

** p<0.001.

**Table 4:** Linear regression predicting depression.

Model	Variable	Unstandardized B	S.E.	Variable p-value	R squared change p-value
1	Highly Traumatized	4.96	0.67	<0.001	<0.001
	Female Gender	1.08	0.50	0.034	
2	Highly Traumatized	3.99	0.64		<0.001
	Female Gender	0.99	0.47		
	External Locus of Control (6-item)	0.44	0.05		
3	Highly Traumatized	3.63	0.65	<0.001	0.046
	Female Gender	1.13	0.47	0.019	
	External Locus of Control (6-item)	0.411	0.05	<0.001	
	Social Support	−0.345	0.12	0.003	
	Age	−0.05	0.12	0.659	
	Married	−1.64	2.47	0.508	
	BMI	0.01	0.04	0.724	
